# Vegetation height and structure drive foraging habitat selection of the lesser kestrel (*Falco naumanni*) in intensive agricultural landscapes

**DOI:** 10.7717/peerj.13979

**Published:** 2022-10-06

**Authors:** Sara Cioccarelli, Anna Terras, Giacomo Assandri, Alessandro Berlusconi, Nunzio Grattini, Alessandro Mercogliano, Aliona Pazhera, Andrea Sbrilli, Jacopo G. Cecere, Diego Rubolini, Michelangelo Morganti

**Affiliations:** 1 Dipartimento di Scienze e Politiche Ambientali, University of Milan, Milan, Italy; 2 Ethology Unit, Department of Biology, University of Pisa, Pisa, Italy; 3 Laboratoire Ecologie et Biologie des Interactions, Université de Poitiers, Poitiers Cedex 9, France; 4 Area Avifauna Migratrice, Istituto Superiore per la Protezione e la Ricerca Ambientale (ISPRA), Ozzano Emilia (BO), Italy; 5 Environment Analysis and Management Unit—Guido Tosi Research Group— Department of Theoretical and Applied Sciences, Università degli Studi dell’Insubria, Varese (VA), Italy; 6 Consiglio Nazionale delle Ricerche—Istituto di Ricerca sulle Acque (CNR-IRSA), Brugherio (MB) and Montelibretti (RM), Italy; 7 SOM Stazione Ornitologica Modenese “Il Pettazzurro”, Mirandola (MO), Italy

**Keywords:** Alfalfa, Agroecosystems, Biodiversity-friendly cultivations, GPS tracking, Harvesting, Habitat selection, Winter cereals

## Abstract

Habitat selection in animals is a fundamental ecological process with key conservation implications. Assessing habitat selection in endangered species and populations occupying the extreme edges of their distribution range, or living in highly anthropized landscapes, may be of particular interest as it may provide hints to mechanisms promoting potential range expansions. We assessed second- and third-order foraging habitat selection in the northernmost European breeding population of the lesser kestrel (*Falco naumanni*), a migratory falcon of European conservation interest, by integrating results obtained from 411 direct observations with those gathered from nine GPS-tracked individuals. The study population breeds in the intensively cultivated Po Plain (northern Italy). Direct observations and GPS data coincide in showing that foraging lesser kestrels shifted their habitat preferences through the breeding cycle. They positively selected alfalfa and other non-irrigated crops during the early breeding season, while winter cereals were selected during the nestling-rearing phase. Maize was selected during the early breeding season, after sowing, but significantly avoided later. Overall, vegetation height emerged as the main predictor of foraging habitat selection, with birds preferring short vegetation, which is likely to maximise prey accessibility. Such a flexibility in foraging habitat selection according to spatio-temporal variation in the agricultural landscape determined by local crop management practices may have allowed the species to successfully thrive in one of the most intensively cultivated areas of Europe. In the southeastern Po Plain, the broad extent of hay and non-irrigated crops is possibly functioning as a surrogate habitat for the pseudo-steppe environment where most of the European breeding population is settled, fostering the northward expansion of the species in Europe. In intensive agricultural landscapes, the maintenance of alfalfa and winter cereals crops and an overall high crop heterogeneity (deriving from crop rotation) is fundamental to accommodate the ecological requirements of the species in different phases of its breeding cycle.

## Introduction

Anthropogenic global change is impacting the environment, either directly by transforming the landscape, as in the case of agricultural intensification, or indirectly through climate change and pollution (Smith et al., 2016). Overall, the growth and expansion of anthropogenic activities are the main causes of habitat loss and fragmentation on a global scale (Fischer & Lindenmayer, 2007). Species living in highly anthropized landscapes, such as intensive agroecosystems, face the challenge of finding habitats that can support their energetic needs (Foley et al., 2005). Studying habitat selection, *i.e.* the disproportionate use of certain habitats compared to their relative availability (Johnson, 1980; Manly et al., 2004), in these species may thus provide key information to understand how they can adapt to such altered habitats. Habitat selection is a hierarchical process that acts at multiple spatial scales: first-order selection represents the selection of the geographical range of a species; second-order selection refers to the home range of an individual within its distribution; third-order selection is defined as the use of different landscape/habitat patches within the home-range and, eventually, fourth-order selection refers to the use of feeding sites within a habitat patch (Johnson, 1980; Meyer & Thuiller, 2006). Specific habitats can be preferred at one scale but not at another: hence, multiscale studies provide a more comprehensive characterization of habitat selection patterns and processes (Mayor et al., 2009).

Not all species have the same ability to tune their habitat preferences according to the local environmental context. Species or populations showing higher degrees of behavioural plasticity are potentially more resilient to environmental changes (Ghalambor et al., 2007). The study of habitat selection in populations occupying the edge of their ecological niche, either in terms of distribution range or landscape context, is of particular interest since it may provide information on the niche breadth of a species (Block & Brennan, 1993; Storch, 2002; Ciucci, Masi & Boitani, 2003). Moreover, habitat selection studies represent a prerequisite to targeted conservation measures that could ensure the long-term persistence of species of conservation priority (Garshelis, 2000) and are a useful baseline for habitat restoration or management interventions (Morrison, 2013; Assandri et al., 2018). Such conservation measures are of special importance to guide conservation actions targeting farmland birds, which are among the group of vertebrates showing the steepest population declines in Europe (Gregory et al., 2019; Burns et al., 2021).

In this study, we investigated foraging habitat selection in the northernmost (45°N) European breeding population of the lesser kestrel (*Falco naumanni*), a small (∼120 g) colonial migratory raptor of conservation priority in the European Union (Iñigo & Barov, 2010), whose breeding range extends to mid-latitudes and low elevation regions in Eurasia and North Africa (Ferguson-Lees & Christie, 2001). This small population has recently settled in the central-eastern Po Plain (northern Italy) (since 2014; La Gioia, Melega & Fornasari, 2017). The Po Plain is characterised by a highly intensively cultivated landscape, almost completely lacking semi-natural habitats such as grasslands and pseudo-steppes, typically preferred in the core of its range in southern Europe (*i.e.* Catry et al., 2012; Morganti et al., 2021).

Unlike previous studies of habitat selection in the lesser kestrel (*e.g.*, Catry et al., 2012; Rodríguez et al., 2012; Rodríguez et al., 2014; Morganti et al., 2021; Assandri et al., 2022; Berlusconi et al., 2022), we relied on two complementary methodologies, visual observation of foraging birds and individual GPS tracking, that allowed us to explore two different orders of habitat selection, the second (home-range) and the third (within home-range), respectively. The collected data were associated with fine-grained habitat variables recorded in the field. Both bird occurrences and habitat data were assessed across three phases of the breeding season (late incubation, early nestling rearing, and late nestling rearing) to investigate possible temporal shifts in the habitat selection performed by lesser kestrels living in highly dynamic farmland landscapes (Catry et al., 2012; Christakis & Sfougaris, 2021; Morganti et al., 2021).

Specifically, we investigated the effects of landscape composition and vegetation structure on foraging site selection. Regarding second-order habitat selection, we explored (1) how lesser kestrels select their foraging home range in the area surrounding the colony and (2) whether this selection changes through the breeding season, as expected based on previous studies (Morganti et al., 2021). With respect to third-order habitat selection, we asked (3) whether vegetation height and structure correlate with the probability of a crop to be used as a foraging (hunting) site and explored (4) whether specific crop types were preferred for foraging. Our ultimate goal was to understand how lesser kestrels cope with such an intensive agroecosystem, which may aid conservation efforts targeting this species.

## Materials & Methods

### Study species

The lesser kestrel is a migratory bird whose European populations spend most of the non-breeding period in the Sahel (Sarà et al., 2019; Sarà et al., 2021). Within the European breeding range, the northernmost limit of the distribution is reached in the eastern Po Plain (Italy, ∼45°N) (La Gioia, Melega & Fornasari, 2017, Fig. 1A), where this study was performed. After a severe population decline experienced during the second half of the 20th century (Iñigo & Barov, 2010), this species became extinct or almost extinct in several countries (*e.g.*, Austria, Hungary, Poland, France, Portugal, Bulgaria) (Bijleveld, 1974). Nowadays, the species has partially recovered, with a European population estimated at 30,500–38,000 pairs (BirdLife International, 2017). It is a species of conservation interest in the European Union (Annex 1, ‘Birds Directive’ 2009/147/CE; Iñigo & Barov, 2010).

**Figure 1:**
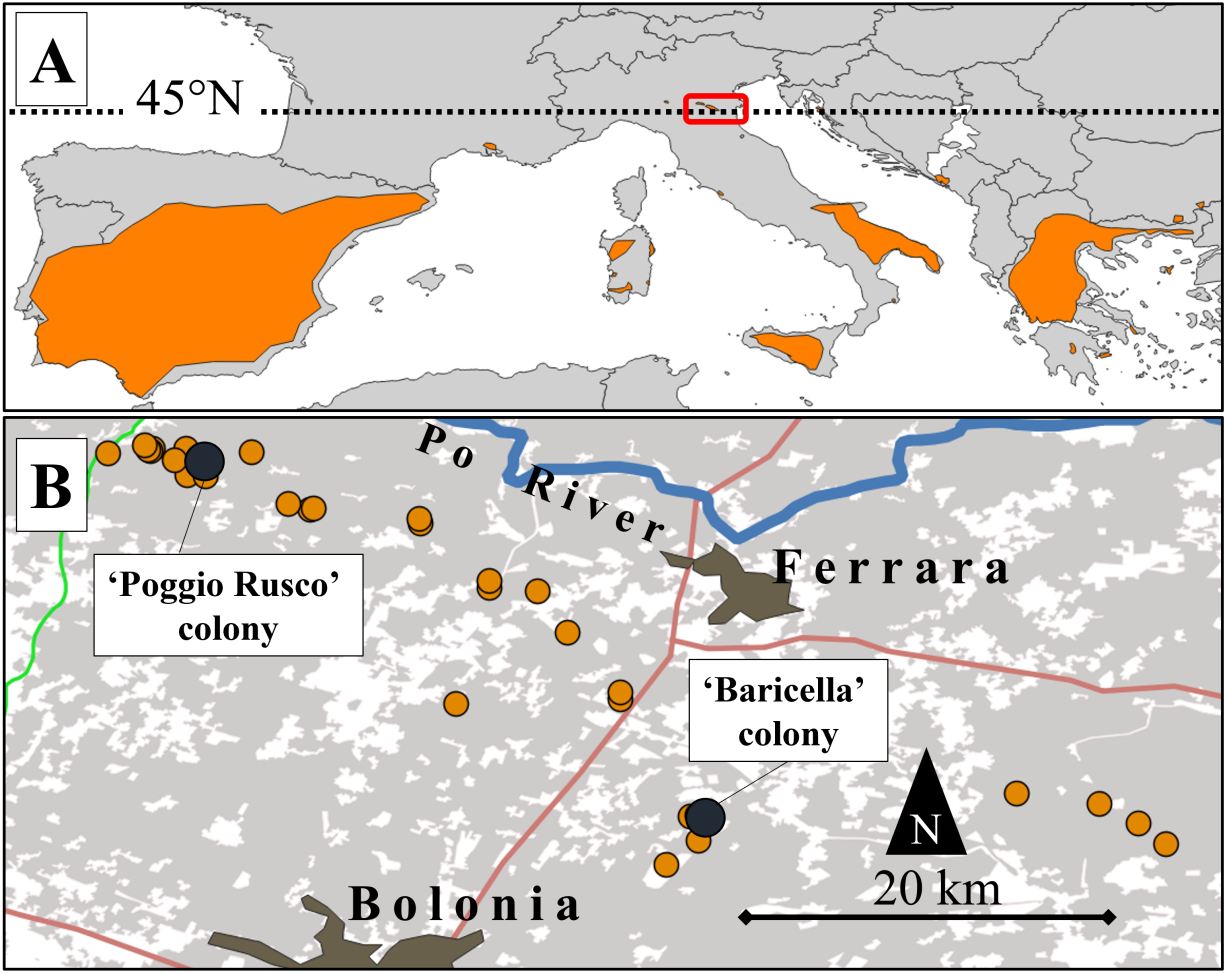
Map of the study area and its location relative to the lesser kestrel distribution range. (A) European distribution of the lesser kestrel (updated to 2019; coloured areas) and location of the study area (red box). (B) Distribution of lesser kestrel colonies (orange dots) in the central-eastern Po Plain in 2019. The two colonies where GPS devices were deployed are highlighted by dark dots. Main roads (red and green lines), the Po River (blue line) and main urban centres are also represented. The grey shaded areas represent the distribution of the non-irrigated crops (mainly winter cereals and alfalfa).

Agricultural transformations of pastures and steppe areas have determined a strong negative impact on lesser kestrel populations (La Gioia, Melega & Fornasari, 2017). This small raptor feeds mostly on large insects, mainly *Orthoptera* and *Coleoptera* (Di Maggio, Campobello & Sarà, 2018), but small vertebrates such as voles and shrews, small passerines, lizards, as well as chilopods may also be consumed (Cramp & Simmons, 1980).

South European lesser kestrel populations are potentially threatened by climate change. On the one hand, the progressive reduction of spring precipitation is shrinking the climatic suitability of the breeding core areas in southern Italy, where ∼15% of the global population of lesser kestrels currently occurs (Morganti, Preatoni & Sarà, 2017). The increase in summer temperatures may push the species beyond its thermal limits (Catry et al., 2015), severely reducing breeding success and triggering population declines (Catry, Franco & Sutherland, 2011). On the other hand, temperature warming may favour the northward expansion of the species. Indeed, the population breeding in north-eastern Po Plain was first detected in 2014, with no previous historical breeding records in the area (La Gioia, Melega & Fornasari, 2017). Hence, the Po Plain could represents a core area where—at least climatically—the species could safely thrive in the near future (Morganti, Preatoni & Sarà, 2017; Berlusconi et al., 2022). The increasing breeding population of the Po Plain could therefore play a key role in the recolonization of formerly lost breeding areas in central Europe. Understanding how this population has adapted to thrive in this intensive agricultural landscape is therefore pivotal to supporting future conservation efforts. In 2018–2021, the overall local population was estimated to be ca. 100–140 pairs (Morganti et al., 2019). The lesser kestrel has shown synanthropic habits for at least the past 2,000 years, either in rural or urban areas (Negro et al., 2020). In the Po Plain, it breeds in small colonies (1–13 pairs) on isolated rural buildings, mostly decaying and abandoned. As in other parts of the breeding range (*e.g.*, Aragón, NE Spain, Ursúa, 2006), the distribution of colonies tends to be spatially aggregated (Fig. 1).

### Study area

We focused on an area of the eastern Po Plain encompassing 16 colonies, that represent all those known up to 2019 and distributed over a large area (∼3,000 km^2^) delimited to the north by the Po River and to the south by the Apennines (Fig. 1B). This wide sector of the Po Plain encompasses lowlands, with a mean altitude of 13 m a.s.l., characterized by a Mediterranean sub-oceanic to sub-continental climate (Costantini, Fantappié & L’Abate, 2013). The mean annual precipitation is 666 mm, while the mean annual temperature is 13.6 °C (climatic data for Ferrara; retrieved from http://www.climate-data.org).

The landscape of the study area is largely cultivated, with only residual natural and semi-natural habitat patches represented by a few inland wetlands and by the Po River wooded riverbanks. The most abundant crops are hayfields of alfalfa *Medicago sativa* and winter cereals (mostly wheat, barley, rye, and triticale), followed by irrigated summer crops (mostly maize, soybean and horticultural crops) (Regione Emilia-Romagna, 2009).

**Figure 2:**
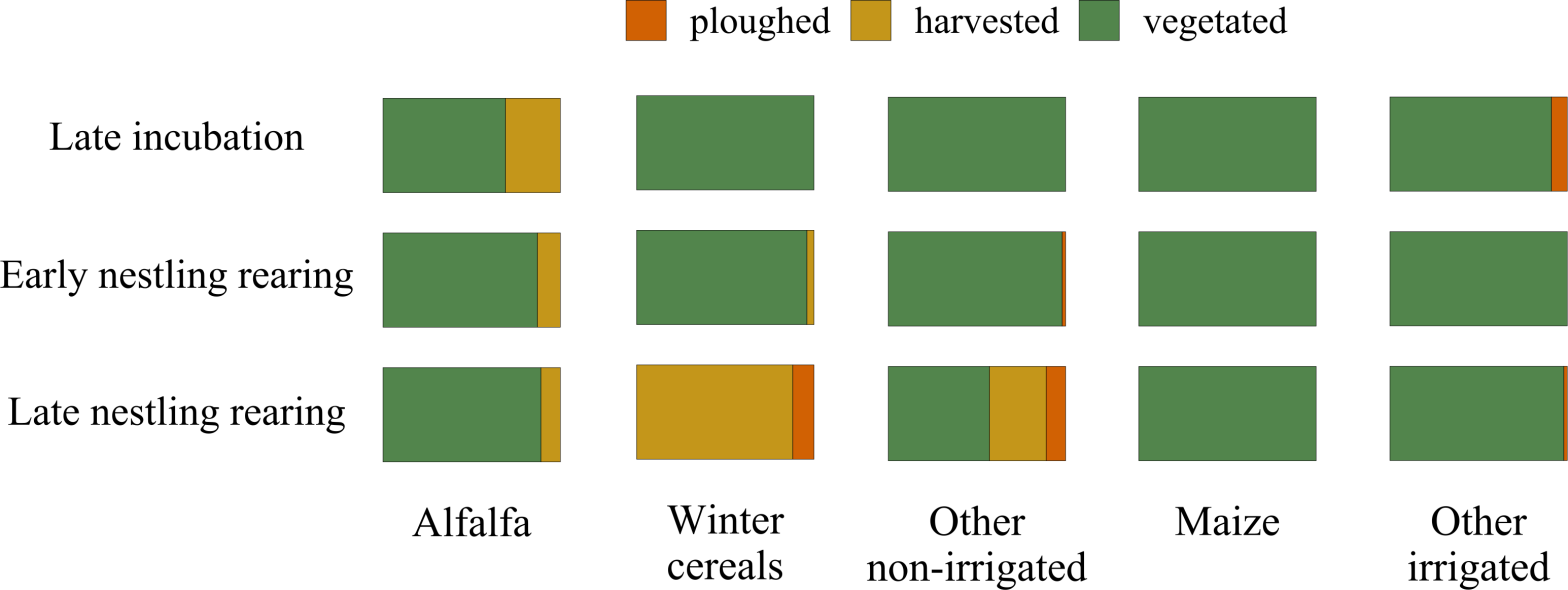
Variation in the cover of vegetation structure categories during the lesser kestrel breeding season. Relative cover of crops according to three vegetation structure categories (ploughed in orange, harvested in yellow and vegetated in green) within the 3 km buffer surrounding breeding colonies where GPS devices were deployed. The overall percentage of each crop within the 3 km buffer is: alfalfa 18%, winter cereal 33%, other non-irrigated crops 3%, maize 8% and other irrigated crops 20%.

Alfalfa crops are harvested up to 4-6 times per season: therefore, during the lesser kestrel breeding season they may appear both vegetated and harvested, and are generally non-irrigated; winter cereals and other non-irrigated crops, by contrast, are sown in autumn, harvested in late spring or early summer and then ploughed. Maize and other summer irrigated crops are sown in spring and harvested at the end of summer, thus are permanently vegetated during the lesser kestrel breeding season. The maize plant height considerably increases from a few centimetres at the beginning of the lesser kestrel breeding season to more than 2 m at its end (Fig. 2).

### GPS tracking and land use data collection

Nine breeding adult lesser kestrels were captured in 2019 (late May-early June, see Table S1) directly in their nesting cavity or using mist-nets at two colony sites of the study area: Poggio Rusco (44.96°N, 11.10°E, hereafter ‘PR’) and Baricella (44.64°N, 11.54°E; hereafter ‘BA’ Fig. 1B). Bird captures and GPS deployments were performed by the Istituto Nazionale per la Protezione e la Ricerca Ambientale (ISPRA) according to the ASAB/ABS guidelines for animal welfare in research, under the authorization of Law 157/1992 (Art. 4 (1) and Art. 7 (5)). Birds were sexed according to plumage (Demongin, 2016). Breeding kestrels were equipped with GPS-UHF loggers (NanoFix GEO + RF, PathTrack Ltd., UK). The devices were deployed with a Teflon wing-loop harness (see details in Cecere et al., 2018). The mass of devices with the harness summed up to (mean ± SD) 4.62 ± 0.18 g, resulting in a relative load of 3.14 ± 0.39% of body mass (range: 2.58–3.90%). The devices were programmed to record one GPS location every 15 min, from sunrise to sunset (3:00–21:00 h UTC; 5–22 h local time). GPS locations were retrieved via UHF using a solar-driven base station. So far, the last contact date is a good proxy of the last day the birds spent in the proximity of the colony, after which they perform post-breeding movements before migration (see Sarà et al., 2014).

Since we aimed at exploring the change in habitat selection through the breeding season, movement data were grouped into three phenological phases, respectively representing late incubation (31 May–15 June), early nestling rearing (16 June–6 July), and late nestling rearing (7–25 July). The incubation lasts on average 28-29 days and is carried out by both individuals (Cramp & Simmons, 1980), the late incubation phase represents the last two weeks of this period. The early nestling rearing phase lasts up to 15 days of age and corresponds to the linear growth phase of nestlings (Podofillini et al., 2018; Romano et al., 2021). The late nestling rearing phase is characterized by the last period of parental care, during which nestlings undergo mass recession and during which fledglings become progressively independent from their parents.

For each phase, we built a detailed map of the landscape composition within a 3 km buffer around the colonies that hosted GPS-tracked birds. For each phase, we additionally assessed the phenological state of each cultivated parcel, *i.e.* ploughed, harvested, or vegetated (Fig. 2). We opted for this buffer size because, during the breeding season, lesser kestrels breeding in rural areas mostly forage within a 3 km radius around the colony (Catry et al., 2013; Catry, Franco & Moreira, 2014; Sarà et al., 2014; Cecere et al., 2018; Assandri et al., 2022). Overall, 51 habitat classes were initially identified in the field, but these were successively merged into 12 to perform analyses and improve the interpretability of the findings (Table S2). Since the mean location error of the GPS was 40 m (Assandri et al., 2022), we rasterized the vectorial map describing the habitat availability at this resolution (*i.e.* raster cell borders of 40 m length).

We retained only those GPS locations belonging to individuals that satisfied the following criteria: (1) had an active nest during the tracking period; (2) had at least 100 valid locations; (3) were successfully tracked for at least 10 days (see Assandri et al., 2022). Furthermore, we excluded all locations belonging to roosting sites, either because they were collected from dusk to dawn (20:00–6:00 h) or because they were within 50 m of the colony site (see Cecere et al., 2018 for a similar approach). For the colony of Poggio Rusco, this buffer was extended to 80 m because the observational data showed that no bird foraged within this distance from the colony. To these buffers, we added a further 40 m due to the location error of the GPS. Overall, we based our analyses on tracking data from two individuals breeding in Poggio Rusco and seven individuals breeding in Baricella, totalling 2,596 and 3,166 GPS locations, respectively (see Table S1 for sample size details).

### Visual observations of foraging attempts and environmental data collection

During both incubation and nestling rearing, male and female lesser kestrels share reproductive duties and move back and forth between the colony and specific foraging areas (*i.e.* behaving as ‘central-place foragers’; Catry et al., 2012; Cecere et al., 2018; Vidal-Mateo, Romero & Urios, 2019, Ramellini et al., 2022). In isolated rural colonies, most of the foraging attempts occur within a few hundred meters from the colony (Cecere et al., 2018; Assandri et al., 2022). These features make it possible to visually study the foraging attempts by concentrating the sampling efforts with the colony as a reference centre. With this aim, we selected a series of vantage points near the colonies, from which to observe the birds without interferring with their foraging activity. Observations of foraging birds were collected during the breeding seasons in 2018 (30 May-28 June) and 2019 (9 May-17 June). The observations were performed in sessions of 30 min by using binoculars, avoiding cold, rainy and windy conditions, when the animals may forage less frequently. It was not possible to identify individuals; therefore, it could be possible that some observations belonged to the same bird.

Data collection was designed to allow a comparison between foraging locations where prey capture was attempted (irrespective of whether it was successful or not) and control locations, paired with the foraging location in all subsequent analyses. The field procedure for the direct observation was arranged in the following steps: (1) an observer identified a lesser kestrel potentially in search of food (lesser kestrels show two main foraging tactics, one more ‘static’ that is perching from a vantage point and one more ‘dynamic’, which involves frequent gliding and hovering; Cecere et al., 2020), (2) when the lesser kestrel made a foraging attempt (*i.e.* diving into the vegetation), the observer recorded the coordinates of that location (the foraging location), (3) obtained a random angle and identified a control location 500 m away from the foraging location in the direction of the random angle, assuming north as 0°, east 90° and so on (Fig. S1 in the online Supplementary Material). Control locations were placed exclusively in habitat typologies potentially suitable for foraging lesser kestrel (*i.e.* avoiding urban areas, paved roads, and orchards). Five hundred meters was considered an adequate distance to position control locations because two locations at this distance are certainly available (*i.e.* accessible) to an actively foraging kestrel but at the same time are likely to fall in two different parcels given the average field size of the study area (ca. 10 ha, details not shown). This approach fits the definition of third-order habitat selection, *i.e.* the selection of different habitat patches within the foraging home-range of a species (Johnson, 1980; Meyer & Thuiller, 2006). (4) Environmental data of both foraging and control locations were collected directly in the field soon after the observation of the foraging attempt (see Figs. S2A–S2C for landscape images of the study area), allowing the assessment of micro-habitat features which can rapidly change as the season progresses. Specifically, vegetation height was measured with a meter directly on the crop; when the crop was not reachable the location was discarded. The data collected for both foraging and control locations were the colony of origin of each bird (birds were attributed to the nearest colony), date and hour, coordinates (latitude and longitude in decimal degrees), vegetation height (cm), vegetation structure (3-levels categorical factor): vegetated (unripe or ripe crops before being harvested), harvested, ploughed (ploughed fields or naturally non-vegetated areas), and type of crop (5-level categorical factor: alfalfa, winter cereals, maize, other irrigated crops, other non-irrigated crops, see Table S2 for a description of the categories). Prey item identity was not recorded as it was impossible to determine in most cases.

Overall, we collected information on 411 foraging observations (259 in 2018, 152 in 2019), and an equal number of control locations. In both years, foraging observations were unevenly distributed among the colonies, with most observations (55% in 2018 and 37% in 2019) belonging to the same colonies where we deployed GPS loggers. Unlike GPS data, visual observation data were not assigned to breeding phases, because the observations made in 2019 belonged almost exclusively to the late incubation phase, and no observations were available in July.

### Statistical analyses

#### Second-order habitat selection analysis

Based on GPS locations, we calculated the individual selection ratio accordingly to a use-vs-availability design, where the selection ratio is defined as the ratio between use and availability measures (Manly et al., 2004). The available habitats were assumed to be the proportional cover of a given habitat class within the 3 km buffer of the colony to which each individual belongs. Analogously, we calculated the use as the ratio between the number of locations falling into each habitat class and the overall number of individual locations within the 3 km buffer. Individual specific selection ratios were calculated for each habitat class and breeding phase. Reliable selection ratios with credible confidence intervals are preferentially calculated for habitat classes in which the number of used items (‘locations’) is greater than four, as recommended in Manly et al. (2004). We respected this rule-of-thumb by only calculating selection ratios for habitat classes that summed up to at least five used locations. However, we verified that repeating the analysis including these underrepresented habitat classes did not qualitatively alter our findings (details not shown).

Selection ratios with their relative 95% confidence intervals (95% CI) were estimated using the Koopman’s score method, with the ‘ci-prat’ function of the ‘asbio’ package (Aho, 2014) as recommended in Aho & Bowyer (2015).

Population-level overall habitat selection was estimated by fitting three separate Generalized Linear Mixed Models (GLMMs) for each phase. The individual selection ratio was entered as the dependent variable in all these models, whereas the habitat category was entered as a fixed categorical factor (eight levels) and, eventually, the grouped structure of the data was accounted for by entering a random factor including the individual identity and the the colony site (Poggio Rusco or Baricella) as random intercept effects. Selection ratios were weighted by including the reciprocal of the confidence interval length as a weight variable (Murtaugh, 2007; Fieberg et al., 2010). GLMMs were fitted with a gamma error distribution and a log-link function, as selection ratios are strictly positive (Zuur, Hilbe & Leno, 2013). This approach is particularly suited for studies based on categorical habitat covariates and allows accounting for individual differences in habitat selection within a population (Assandri et al., 2022). GLMMs were fitted using the R-package ‘glmmTMB’ ver. 1.1.2.3 (Brooks et al., 2017). The significance of fixed factors was tested using the Wald chi-square test using the ‘car’ R-package ver. 3.0-12 (Fox & Weisberg, 2019), collinearity was tested using R-package ‘misty’ ver. 0.4.4 (Yanagida, 2022) and model assumptions were checked using the ‘DHARMa’ R-package ver. 0.4.4 (Hartig, 2021).

#### Third-order habitat selection analysis

To assess which factors were influencing the probability of a field to be used as a foraging location by lesser kestrels, we relied on conditional logistic regression, comparing the habitat features of foraging vs. control locations in a pairwise framework (Garshelis, 2000). Data collection was structured to record, for each single foraging and control location, the vegetation height, the vegetation structure, and the crop type of the field where the location was falling. These predictors were not independent: vegetation height (continuous variable) was not independent from the vegetation structure or crop type. For this reason, we fitted three separate models to explore the effects of (1) vegetation height, (2) habitat structure and (3) crop type on the probability of a field being used as a foraging location. Conditional logistic regression models had a common structure: the response variable was binary (foraging vs. control location) and the identifier of each pair of data was used as a stratifying factor, to maintain the pairwise structure of the comparisons. In the first model, the vegetation height was entered as a continuous predictor. In the second model, the predictor was a categorical variable expressing vegetation structure (three levels: vegetated, harvested, ploughed). The third model was aimed to disentangle, among vegetated crops, if the lesser kestrels selected specific crop types: we thus fitted a model with a 5-level categorical variable (*i.e.* alfalfa, winter cereals, other non-irrigated crops, maize, other irrigated crops) as a predictor. Conditional logistic regression models were fitted in the ‘survival’ R-package ver. 3.2-13 (Therneau, 2015). Finally, for each of the conditional logistic regression models, we calculated McFadden’s pseudo-R^2^, defined by the following formula:

\begin{eqnarray*}{\text{pseudo-}R}^{2}=1- \frac{\mathrm{log}({L}_{c})}{\mathrm{log}({L}_{\mathrm{null}})} \end{eqnarray*}

where *L*_*c*_ is the likelihood function for the model being estimated and *L*_null_ is the likelihood for a model with no predictor (McFadden, 1973). This kind of pseudo-R^2^ is a measure of the goodness-of-fit of the model suited for logistic regression models. This pseudo-R^2^ can be used to compare the variance explained by different models that are based on the same dataset. All the analyses were run in R 4.0.2 (R Core Team, 2019).

## Results

### Second-order habitat selection analysis

**Figure 3:**
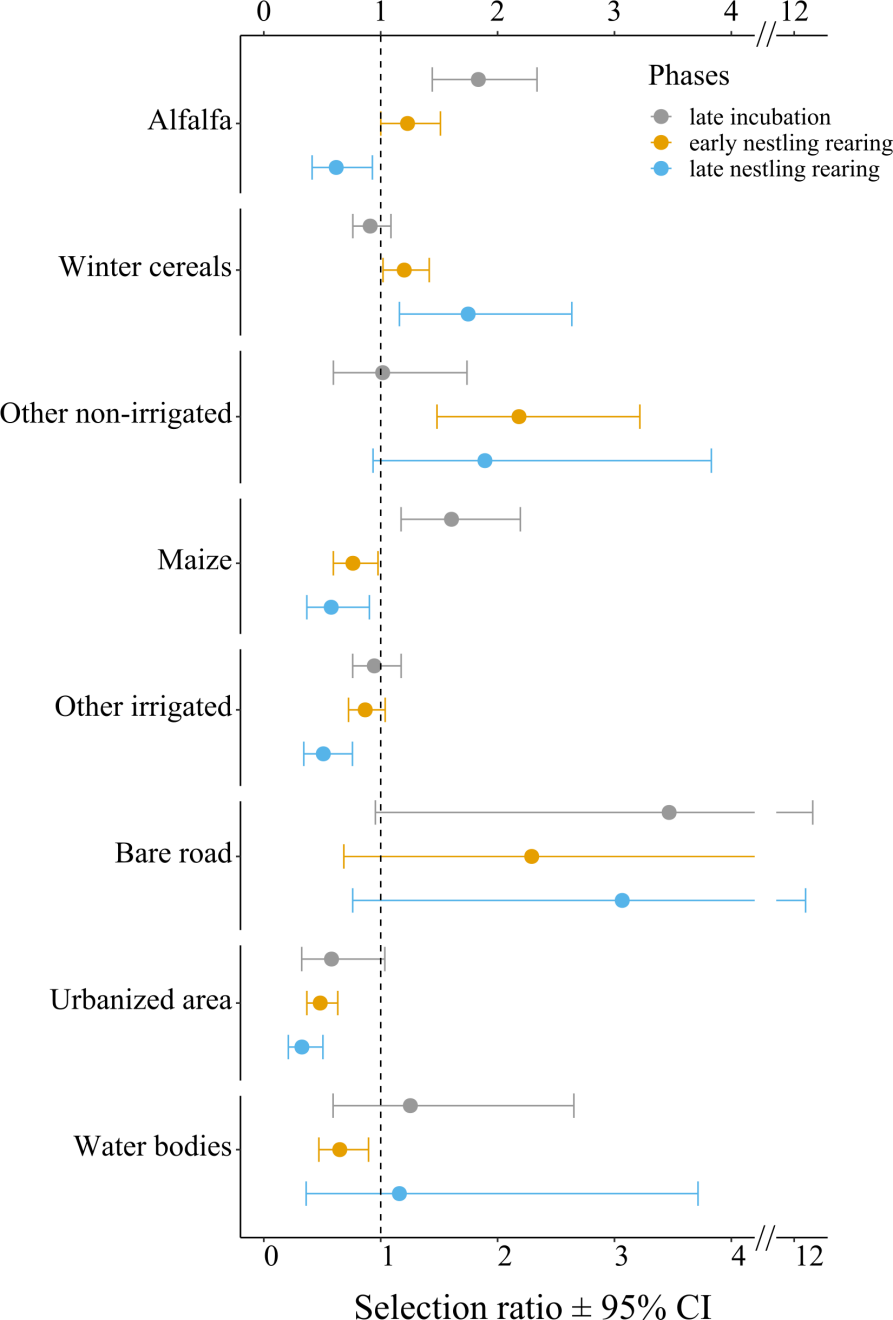
Selection ratios for different crop types during the lesser kestrel breeding phases, obtained from GPS tracking data (*N* = 9 individuals). A selection ratio of 1 (or 95% CI crossing the dashed line) means that no significant selection occurs for a given habitat. A selection ratio above 1 (with 95% CI not encompassing the dashed line) implies that the habitat is significantly positively selected, whereas values below 1 (with 95% CI not encompassing the dashed line) implies that the habitat is avoided.

At the population level, lesser kestrels used habitats differently from what was expected by chance during each breeding phase (late incubation: *χ*^2^ = 43.91, *df* = 7, *p* < 0.001, Nakagawa’s marginal *R*^2^ = 0.63; early nestling rearing phase: *χ*^2^ = 66.97, *df* = 7, *p* < 0.001, *R*^2^ = 0.58; late nesting rearing: *χ*^2^ = 97.90, *df* = 7, *p* < 0.001, *R*^2^ = 0.58). Specifically, during the late incubation, birds significantly and positively selected alfalfa and maize, while avoiding more urbanized areas (*e.g.*, urban areas and other infrastructures, Fig. 3, Table S3A). During the early nestling rearing phase birds positively and significantly selected alfalfa, winter cereals and other non-irrigated crops, while avoiding maize, other irrigated crops, urbanized areas, and water bodies (Fig. 3, Table S3B). Finally, in the late nestling rearing phase, birds positively selected winter cereals and other non-irrigated crops, while avoiding alfalfa, maize, other irrigated crops, and urbanized areas (Fig. 3, Table S3C).

### Third-order habitat selection analysis

**Figure 4:**
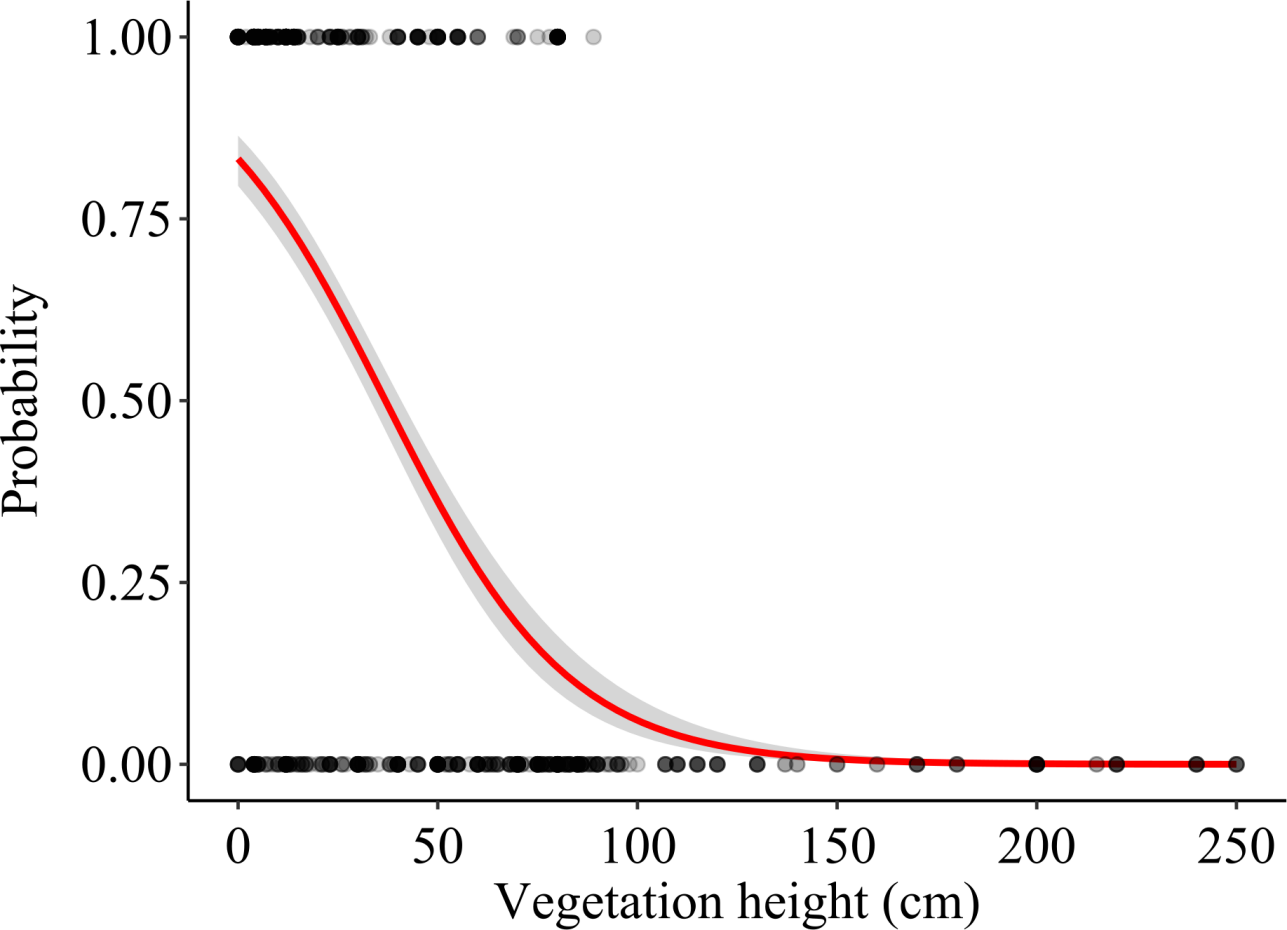
Effects of vegetation height on the probability that a field was used as a foraging location. The logistic regression line (with 95% CI) is shown. Actual foraging and control locations are shown as overlying dots (darker areas corresponding to higher density of overlapping dots).

Vegetation height (mean ± S.E.) was 20.41 ± 1.05 cm at foraging locations and 71.39 ± 2.80 cm at control locations (*N* = 411 pairs). The vegetation height model showed that vegetation height significantly predicted the probability of a field being used as a foraging location, lower vegetation height being favoured (*p* < 0.001; Table 1, Fig. 4). The model assessing the effect of vegetation structure (three categories) was also highly significant (*χ*^2^ = 198.95, *df* = 2, *p* < 0.001), harvested fields being preferred as foraging locations over both vegetated and ploughed crops, whereas no differences in selection occurred between ploughed and vegetated fields (Table 1). Also crop type significantly affected habitat selection (*χ*^2^ = 95.61, *df* = 4, *p* < 0.001); among vegetated crops, alfalfa and other non-irrigated crops were significantly preferred over winter cereals, maize and other irrigated crops as foraging locations. Additionally, winter cereal crops were significantly preferred over maize and other irrigated crops (Table 1). The rest of the pair-wise comparisons were non-significant. The comparison of pseudo-R^2^ between the models suggested that the effect of vegetation height was larger than both the vegetation structure and crop type in predicting the probability of a field being used for foraging and that the effect of vegetation structure was also larger than that of crop type (Table 1).

**Table 1:** Third-order habitat selection: effect of vegetation height and vegetation structure on foraging probability in the lesser kestrel.

**Factors/levels**	**Estimate (±S.E.)**	***z***	***p***
*Vegetation height model* (*pseudo*-*R*^2^ = 0.51)			
Vegetation height	−0.06 (±0.01)	−8.89	**<0.001**
*Vegetation structure model* (*pseudo*-*R*^2^ = 0.35)			
Harvested vs. vegetated	−3.99 (±0.58)	−6.85	**<0.001**
Harvested vs. ploughed	−3.58 (±1.08)	−3.31	**<0.001**
Ploughed vs. vegetated	−0.41 (±0.91)	−0.44	0.657
*Crop type model* (*pseudo*-*R*^2^ = 0.17)			
Alfalfa vs. winter cereals	−0.76 (±0.19)	−4.13	**<0.001**
Alfalfa vs. other irrigated crops	−1.94 (±0.36)	−5.32	**<0.001**
Alfalfa vs. maize	−2.06 (±0.31)	−6.65	**<0.001**
Winter cereals vs. other irrigated crops	−1.17 (±0.35)	−3.36	**<0.001**
Winter cereals vs. maize	−1.30 (±0.31)	−4.24	**<0.001**
Other non-irrigated crops vs. alfalfa	−0.41 (±0.43)	−0.94	0.346
Other non-irrigated crops vs. winter cereals	−1.17 (±0.43)	−2.75	**0.006**
Other non-irrigated vs. other irrigated crops	−2.34 (±0.51)	−4.58	**<0.001**
Other non-irrigated crops vs. maize	−2.47 (±0.50)	−4.98	**<0.001**
Other irrigated crops vs. maize	−0.13 (±0.42)	−0.30	0.765

**Notes.**

Results from two conditional logistic regression models testing for the probability of a field being used as a foraging location according to vegetation height and vegetation structure. For the habitat structure, both the overall significance of the categorical variables and the pair-wise comparison of each level are reported. Significant results are shown in bold. *N* = 411 foraging locations and 411 control locations.

## Discussion

Our study investigated the foraging habitat selection in a lesser kestrel population settled in the intensively cultivated agricultural landscapes of northern Italy (Po Plain), at the northernmost margin of the current European breeding range of the species. We considered two levels of habitat selection; at the second-order, we assessed the foraging selection at the population level in three different phases of the breeding season. At the third-order, we mainly focused on fine-scale habitat features (*i.e.* vegetation height and structure). The investigation over two levels of habitat selection with two complementary approaches allowed us to obtain a complete overview of the spatial complexity of the foraging habitat selection process and of its dynamicity over the lesser kestrel breeding season.

We relied on two complementary approaches: GPS telemetry and direct observations of foraging individuals. Our telemetry data allowed us to investigate the second-order habitat selection at the population level, and to add a temporal component to the foraging selection analysis (*i.e.* to investigate how the foraging habitat selection changed during the breeding season). We found that during incubation, lesser kestrels positively selected alfalfa hayfields and maize. Both these crops, during this phase, present a low vegetation height, although for contrasting reasons. Alfalfa is the first crop to be harvested. Thus, at the end of the spring, a consistent part of hayfields is being harvested or has just been harvested (31% in 2019; Fig. 2). In contrast, maize has just started its growing season, presenting short plants and high availability of bare soil. Later, during the early nestling rearing phase, birds preferentially select alfalfa, which is harvested several times during the season and thus continues to provide favourable foraging habitats, altogether with winter crops and other non-irrigated crops, which begin to be harvested at this time of the year (Fig. 2). Eventually, in the late nestling rearing phase, we found again a strong selection for winter cereals and other non-irrigated crops. This period of the year coincides with the peak of harvesting of those crops (respectively 88% and 32%), which are in some cases ploughed (respectively 12% and 11%, Fig. 2), providing again favourable foraging habitats for the species. Notably, in this phase, alfalfa is not preferred, probably because in most fields it is regrowing and is too high to be suitable for foraging kestrels (Fig. 2), with winter crops representing a more profitable foraging habitat. With the exception of the first phase, maize is significantly avoided. Other irrigated crops (*e.g.*, soybean) were used accordingly to their availability in the first two phases, and then avoided; in addition, in late summer its vegetation height is likely too high for foraging kestrels. Overall, these results suggest that lesser kestrels can track foraging resources both in space and time in a strongly human-modified habitat. This is consistent with what we observed in southern Italy, where non-irrgated crops (cereals) were especially favoured during the mid- and late-breeding season (Morganti et al., 2021; Assandri et al., 2022). We acknowledge that a caveat of our study is the limited number of tracked birds; however, to support the robustness of our findings based on telemetry data, we stress the consistency of these data with previous studies based on similar approaches and performed with larger sample sizes and/or across different populations (Assandri et al., 2022).

Direct observations allowed us to obtain a better understanding of fine-scale foraging habitat selection, suggesting that lesser kestrels tend to prefer low vegetation crop height. Low vegetation height represented the most important factor determining the probability of using a field as a foraging location compared to vegetation structure mediated by management practices (harvesting, ploughing), and, most importantly, crop type.

Foraging habitat selection of lesser kestrels has been previously assessed in other sectors of the lesser kestrel’s breeding range characterized by a lower level of agricultural intensification. Collectively, all studies agree in showing that during in the late breeding season lesser kestrels prefer to forage in harvested fields (*e.g.*, Catry et al., 2012; Catry, Franco & Moreira, 2014; Rodríguez et al., 2014; Morganti et al., 2021). These may include alfalfa, winter cereals, legumes or artichokes, while they systematically avoid wooded areas (*e.g.*, orchards and woodlots) and maize fields. Maize fields are an irrigated crop, fairly widespread not only in the Po Plain but also in other breeding areas of the species, where large areas of non-irrigated agroecosystems were converted to irrigated ones (Franco, Marques & Sutherland, 2005; García et al., 2006; Catry et al., 2012; Catry et al., 2013; Catry, Franco & Moreira, 2014; Rodríguez et al., 2014; Di Maggio, Campobello & Sarà, 2018). The preference for harvested fields may reflect a greater prey availability in these areas: the availability of large orthopterans indeed peaks during and immediately after the harvesting, but persists at relatively high levels for weeks in the post-harvest stubble (Donázar et al., 1993; Catry, Franco & Moreira, 2014), which may explain the selection for harvested fields.

Currently, the core of the European lesser kestrel population occurs in pseudo-steppe landscapes, where non-irrigated crops are scattered within a matrix of semi-natural grasslands or fallow fields. In those contexts, lesser kestrels tend to forage in semi-natural grassland, especially at the beginning of the breeding season, when high energy resources are needed (Catry et al., 2013; Catry, Franco & Moreira, 2014; Morganti et al., 2021; Assandri et al., 2022). In the intensively cultivated Po Plain, semi-natural grasslands are lacking. The observed positive selection for alfalfa, in particular at the beginning of the breeding season, suggests that this crop type may represent a functional replacement for semi-natural grasslands. Preliminary insect and mammal sampling realized in 2021 suggested that alfalfa crops may offer more potential preys compared to other crops, *e.g.*, maize (A. Berlusconi et al, pers. obs., 2021), possibly resembling those of the natural grasslands.

To date, in northern Italy, the lesser kestrel colonized the south-eastern sector of the Po Plain only (Berlusconi et al., 2022). Unlike the rest of the floodplain, in which maize cultivation represents almost 90% of cultivated land, the southeastern plain is characterized by an important proportion of hay crops (mostly alfalfa), aimed at producing fodder for the *Parmigiano Reggiano* cheese supply chain (Mantovi et al., 2015; Battini et al., 2016). This high-quality Protected Designation of Origin cheese can be produced only in the southeastern Po Plain between Parma and Bolonia, and the supply chain for his production has contributed to maintaining the traditional crop rotation which increases the landscape compositional heterogeneity and the availability of suitable crop typologies for the lesser kestrel during the entire breeding season. Indeed, alfalfa and winter cereals proved to be essential to support lesser kestrels during the breeding period; therefore, landscape planning strategies aimed at favouring the lesser kestrels, and likely other farmland birds with similar ecological requirements, should take into account the importance of these crop types (G. Assandri et al., 2022, unpubl. data).

## Conclusions

Our findings have many elements in common with previous studies but provide new insights into the ecology of the lesser kestrel in a novel environmental context, the highly intensively cultivated Po Plain of northern Italy, where the traditional landscape associated with the presence of the species (natural pseudo-steppe grasslands) is lacking. Alfalfa hayfields, other non-irrigated crops and winter cereal crops were often selected as foraging sites because of their vegetation structure and cultivation features. Overall, we found that vegetation height and structure strongly influenced foraging habitat selection, more than crop types. When vegetation height is low, and fields have been harvested, the probability of occurrence of a foraging attempt is likely to be maximized. The investigation over two levels of habitat selection with two complementary approaches was able to provide a broad overview of the complexity of the foraging habitat selection and of its dynamics over the breeding season. Our findings should be considered when planning conservation initiatives for the lesser kestrel in the Po Plain as well as in other intensively cultivated areas of its (northward expanding) breeding range. To obtain an exhaustive understanding of which crops play an important role for the lesser kestrel over its entire breeding cycle, it would be necessary to extend the foraging and habitat selection analyses to the pre- and post-breeding periods. This would provide more comprehensive knowledge to foster the long-term conservation of the lesser kestrel, as well as of other species with similar ecological requirements, in intensively cultivated agroecosystems.

##  Supplemental Information

10.7717/peerj.13979/supp-1 Table S1 Summary information of GPS tracking data for each individual in the 3km buffer of the colony included in the telemetry study, year 2019 Colony: PR Poggio Rusco, BA Baricella. Dates: range of date with tracking data considered in the study. Locations: number of GPS locations collected in total (Overall N) and divided by the three different phases (Late incubation, phase 1; Early rearing, phase 2; Late rearing, phase 3).

10.7717/peerj.13979/supp-2 Table S2 Description of the habitat classes used both for observations of foraging individuals and for telemetry data collection Note that habitat data collected during the observations of foraging individuals only include habitats that were actually used to forage (foraging locations). We explicitly avoided collecting control locations in habitats that were completely unsuitable for the lesser kestrel foraging (*e.g.* wooded patches, water bodies). In contrast, the habitat mapping in the area around the colonies where the telemetry study was based completely covered a buffer of 3 km radius, thus including all the habitat categories truly occurring at the landscape scale.

10.7717/peerj.13979/supp-3 Table S3 Habitat selection through the breeding stages For each habitat class, it is reported: the number of real locations, the habitat availability in hectares, the Manly’s selection ratio and the 95% confidence intervals of the ratio (calculated with Kooper method). A: Late incubation, phase 1, B: Early rearing, phase 2, C: Late rearing, phase 3. See Table S1 for details on each habitat class.

10.7717/peerj.13979/supp-4 Figure S1 Schematic representation of the procedure for observing forage individuals The observer identified a lesser kestrel potentially in search of food from a vantage point near a colony. When a lesser kestrel made a foraging attempt, the observer recorded the coordinates of the foraging location (green dot). Then, given a random angle, the control location (red dot) was assigned to the location falling 500 m away in the direction of the random angle (centred in the foraging location), assuming north as 0°, east 90° and so on.

10.7717/peerj.13979/supp-5 Figure S2 Landscape of the study area (central-eastern Po Plain, northern Italy, Modena-Mantua) A: Winter cereals crops, June 2021. B: Alfalfa crops, June 2019. C: The first building colonized by lesser kestrels in the area. The renovation (April 2018) made it unsuitable for the species to breed. Photo credits: authors of the paper, LIFE FALKON archive.

10.7717/peerj.13979/supp-6 File S1 Foraging observations dataset

10.7717/peerj.13979/supp-7 File S2 Movement dataset

10.7717/peerj.13979/supp-8 File S3 Mapped habitat patches in 3 km radius around the colony for each phase

## Additional Information and Declarations

### Data Availability

The following information was supplied regarding data availability: The raw data are available in the Supplementary Files.
