# Supplementary material for: Vegetation height and structure drive foraging habitat selection of the lesser kestrel (Falco naumanni) in intensive agricultural landscapes
Source: PeerJ. 2022 Oct 6;10:e13979. doi: 10.7717/peerj.13979 (PMC9548312; doi:10.7717/peerj.13979)
Supplement: Table S1 — Colony: PR Poggio Rusco, BA Baricella. Dates: range of date with tracking data considered in the study. Locations: number of GPS locations collected in total (Overall N) and divided by the three different phases (Late incubation, phase 1; Early rearing, phase 2; Late rearing, phase 3). [file peerj-10-13979-s001.docx]

**Table S1:**

**Summary information of GPS tracking data for each individual in the 3km buffer of the colony included in the telemetry study, year 2019.**

Colony: PR Poggio Rusco, BA Baricella. Dates: range of date with tracking data considered in the study. Locations: number of GPS locations collected in total (Overall N) and divided by the three different phases (Late incubation, phase 1; Early rearing, phase 2; Late rearing, phase 3).

|  |  |  | |  |  | | **GPS locations** | | | |
| --- | --- | --- | --- | --- | --- | --- | --- | --- | --- | --- |
| **Bird identity** | **Colony** | | **Sex** | **Dates** | **Days tracked** | **Overall N** | | **Locations**  **phase 1** | **Locations phase 2** | **Locations phase 3** |
| 40512 | PR | M | | 31/05 - 25/07 | 56 | | 1802 | 273 | 925 | 604 |
| 40513 | PR | F | | 31/05 - 21/07 | 52 | | 794 | 159 | 333 | 302 |
| 40516 | BA | F | | 31/05 - 25/07 | 55 | | 593 | 150 | 157 | 286 |
| 40519 | BA | M | | 31/05 - 25/07 | 56 | | 779 | 91 | 321 | 367 |
| 40524 | BA | F | | 31/05 - 22/07 | 53 | | 452 | 115 | 120 | 217 |
| 40525 | BA | M | | 09/06 - 25/07 | 47 | | 740 | 33 | 145 | 562 |
| 40530 | BA | M | | 08/06 - 18/06 | 11 | | 101 | 48 | 53 | 0 |
| 40533 | BA | M | | 11/06 - 25/07 | 45 | | 180 | 15 | 100 | 65 |
| 40535 | BA | F | | 11/06 - 25/07 | 45 | | 321 | 10 | 121 | 190 |
