# Supplementary material for: Vegetation height and structure drive foraging habitat selection of the lesser kestrel (Falco naumanni) in intensive agricultural landscapes
Source: PeerJ. 2022 Oct 6;10:e13979. doi: 10.7717/peerj.13979 (PMC9548312; doi:10.7717/peerj.13979)
Supplement: Table S2 — Note that habitat data collected during the observations of foraging individuals only include habitats that were actually used to forage (foraging locations). We explicitly avoided collecting control locations in habitats that were completely unsuitable for the lesser kestrel foraging (e.g. wooded patches, water bodies). In contrast, the habitat mapping in the area around the colonies where the telemetry study was based completely covered a buffer of 3 km radius, thus including all the habitat categories truly occurring at the landscape scale. [file peerj-10-13979-s002.docx]

**Table S2:**

**Description of the habitat classes used both for observations of foraging individuals and for telemetry data collection.**

Note that habitat data collected during the observations of foraging individuals only include habitats that were actually used to forage (foraging locations). We explicitly avoided collecting control locations in habitats that were completely unsuitable for the lesser kestrel foraging (e.g. wooded patches, water bodies). In contrast, the habitat mapping in the area around the colonies where the telemetry study was based completely covered a buffer of 3 km radius, thus including all the habitat categories truly occurring at the landscape scale.

| **Habitat class** | **Description of the category** | **Observations of foraging individuals** | **Telemetry** |
| --- | --- | --- | --- |
| Alfalfa | Alfalfa *(Medicago sativa)* crops at any growth stage | X | X |
| Ploughed | Ploughed fields | X |  |
| Bare road | Dirt roads |  | X |
| Harvested | Harvested field of any type: alfalfa, barley (*Hordeum vulgare*), coriander (*Coriandrum sativum*), fallow, peas (*Pisum sativum*), rapeseed (*Brassica napus*), rye (*Secale cereale*), triticale (× *Triticosecale*) and wheat (*Triticum spp*.) | X |  |
| Other irrigated crops | Sugar beet (*Beta vulgaris*), melon *(Cucumis melo*), onion (*Allium cepa*), potatoes (*Solanum tuberosum*), pumpkin (*Cucurbita spp*.), sorghum (*Sorghum vulgare*), soy (*Glycine max*), sunflower (*Helianthus annuus*), thistle (*Cynara cardunculus*), tobacco (*Nicotiana tabacum*) and tomatoes (*Solanum lycopersicum*,) | X | X |
| Maize | Maize (*Zea mais*) | X | X |
| Other non- irrigated crops | Chickpeas (*Cicer arietinum*), chicory (*Cichorium intybus*), coriander, fallow, peas and rapeseed | X | X |
| Trees | Fruit orchards, poplar plantations, isolated trees and tree rows, vineyards and woods |  | X |
| Urbanized areas | Greenhouse, isolated buildings, solar panels, streets, urban areas and other infrastructures |  | X |
| Water | Ponds, irrigation canals and ditches, wetlands |  | X |
| Watercourses vegetation | Vegetation surrounding irrigation canals |  | X |
| Winter cereals | Winter cereals are barley, rye, triticale, and wheat | X | X |
