# Supplementary material for: Vegetation height and structure drive foraging habitat selection of the lesser kestrel (Falco naumanni) in intensive agricultural landscapes
Source: PeerJ. 2022 Oct 6;10:e13979. doi: 10.7717/peerj.13979 (PMC9548312; doi:10.7717/peerj.13979)
Supplement: Table S3 — For each habitat class, it is reported: the number of real locations, the habitat availability in hectares, the Manly’s selection ratio and the 95% confidence intervals of the ratio (calculated with Kooper method). A: Late incubation, phase 1, B: Early rearing, phase 2, C: Late rearing, phase 3. See Table S1 for details on each habitat class. [file peerj-10-13979-s003.docx]

**Table S3:**

**Habitat selection through the breeding stages.**

For each habitat class the Manly’s selection ratio and the 95% confidence intervals of the ratio (estimated by GLMMs). **A:** Late incubation, phase 1, **B:** Early rearing, phase 2, **C:** Late rearing, phase 3. See Table S1 for details on each habitat class.

**A: Late incubation, phase 1**

| **Habitat** | **Selection ratio** | **Lower 95% CI** | **Upper 95% CI** |
| --- | --- | --- | --- |
| Alfalfa | 1.84 | 1.44 | 2.34 |
| Winter cereals | 0.91 | 0.76 | 1.09 |
| Other non-irrigated | 1.02 | 0.60 | 1.74 |
| Maize | 1.61 | 1.17 | 2.20 |
| Other irrigated | 0.95 | 0.76 | 1.18 |
| Bare road | 3.47 | 0.95 | 12.60 |
| Urbanized area | 0.45 | 0.20 | 0.98 |
| Water Bodies | 1.33 | 0.60 | 2.99 |

**B: Early rearing, phase 2**

| **Habitat** | **Selection ratio** | **Lower 95% CI** | **Upper 95% CI** |
| --- | --- | --- | --- |
| Alfalfa | 1.23 | 1.00 | 1.51 |
| Winter cereals | 1.20 | 1.02 | 1.42 |
| Other non-irrigated | 2.18 | 1.48 | 3.22 |
| Maize | 0.76 | 0.59 | 0.98 |
| Other irrigated | 0.87 | 0.72 | 1.04 |
| Bare road | 2.29 | 0.68 | 7.67 |
| Urbanized area | 0.48 | 0.37 | 0.63 |
| Water Bodies | 0.65 | 0.47 | 0.90 |

**C: Late rearing, phase 3**

| **Habitat** | **Selection ratio** | **Lower 95% CI** | **Upper 95% CI** |
| --- | --- | --- | --- |
| Alfalfa | 0.62 | 0.41 | 0.93 |
| Winter cereals | 1.75 | 1.16 | 2.64 |
| Other non-irrigated | 1.89 | 0.93 | 3.83 |
| Maize | 0.58 | 0.37 | 0.90 |
| Other irrigated | 0.51 | 0.34 | 0.76 |
| Bare road | 3.07 | 0.76 | 12.37 |
| Urbanized area | 0.33 | 0.21 | 0.51 |
| Water bodies | 1.16 | 0.36 | 3.71 |
