# Supplementary figures and images for: Vegetation height and structure drive foraging habitat selection of the lesser kestrel (Falco naumanni) in intensive agricultural landscapes
Source: PeerJ. 2022 Oct 6;10:e13979. doi: 10.7717/peerj.13979 (PMC9548312; doi:10.7717/peerj.13979)

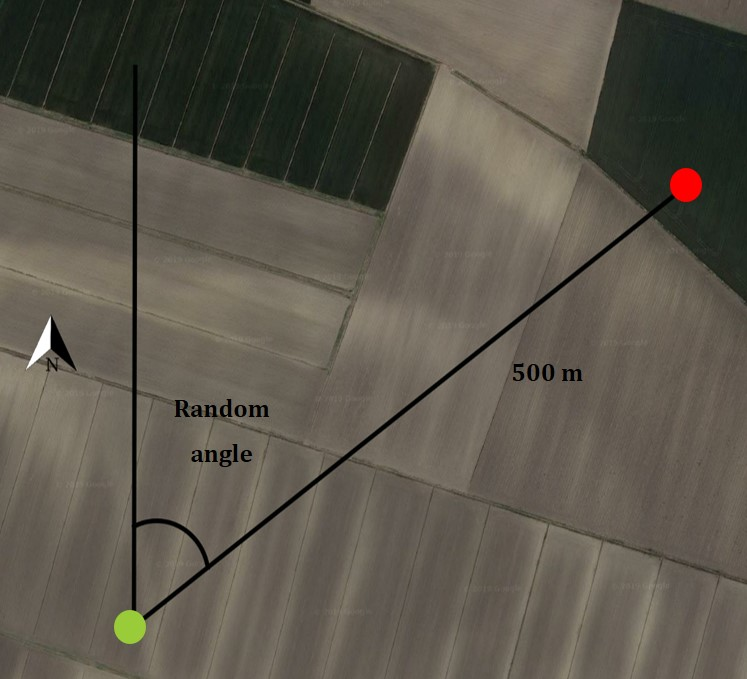

Supplement: Figure S1 — The observer identified a lesser kestrel potentially in search of food from a vantage point near a colony. When a lesser kestrel made a foraging attempt, the observer recorded the coordinates of the foraging location (green dot). Then, given a random angle, the control location (red dot) was assigned to the location falling 500 m away in the direction of the random angle (centred in the foraging location), assuming north as 0°, east 90° and so on. [file peerj-10-13979-s004.png]

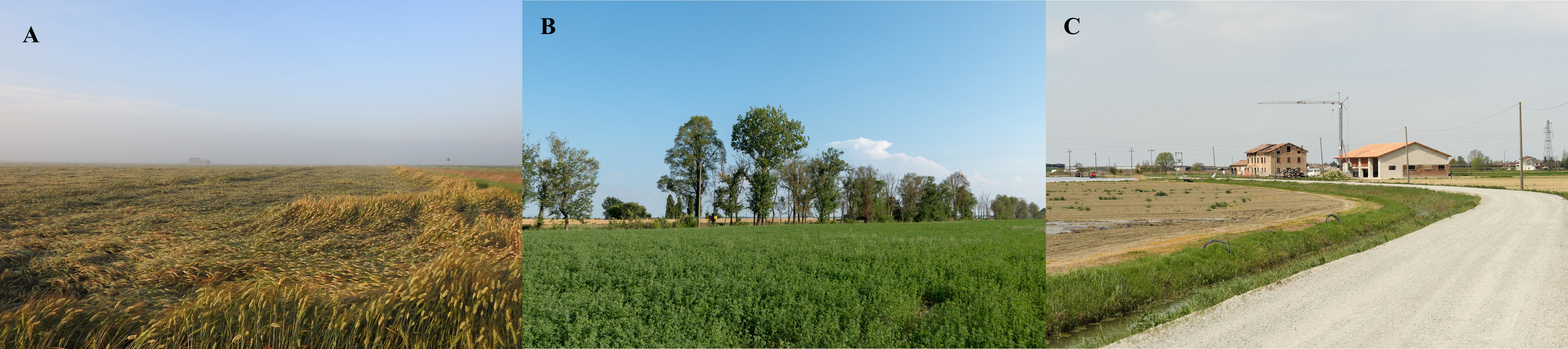

Supplement: Figure S2 — A: Winter cereals crops, June 2021. B: Alfalfa crops, June 2019. C: The first building colonized by lesser kestrels in the area. The renovation (April 2018) made it unsuitable for the species to breed. Photo credits: authors of the paper, LIFE FALKON archive. [file peerj-10-13979-s005.png]
